# Meet-U: Educating through research immersion

**DOI:** 10.1371/journal.pcbi.1005992

**Published:** 2018-03-15

**Authors:** Nika Abdollahi, Alexandre Albani, Eric Anthony, Agnes Baud, Mélissa Cardon, Robert Clerc, Dariusz Czernecki, Romain Conte, Laurent David, Agathe Delaune, Samia Djerroud, Pauline Fourgoux, Nadège Guiglielmoni, Jeanne Laurentie, Nathalie Lehmann, Camille Lochard, Rémi Montagne, Vasiliki Myrodia, Vaitea Opuu, Elise Parey, Lélia Polit, Sylvain Privé, Chloé Quignot, Maria Ruiz-Cuevas, Mariam Sissoko, Nicolas Sompairac, Audrey Vallerix, Violaine Verrecchia, Marc Delarue, Raphael Guérois, Yann Ponty, Sophie Sacquin-Mora, Alessandra Carbone, Christine Froidevaux, Stéphane Le Crom, Olivier Lespinet, Martin Weigt, Samer Abboud, Juliana Bernardes, Guillaume Bouvier, Chloé Dequeker, Arnaud Ferré, Patrick Fuchs, Gaëlle Lelandais, Pierre Poulain, Hugues Richard, Hugo Schweke, Elodie Laine, Anne Lopes

**Affiliations:** 1 Departments of Computer Science and of Life Sciences, Sorbonne Université (SU) / UPMC, Paris, France; 2 Department of Biology and of Computer Science, Univ. Paris-Sud, Université Paris-Saclay (UPSay), Orsay, France; 3 Unit of Structural Dynamics of Macromolecules, CNRS, Institut Pasteur, Paris, France; 4 Institute for Integrative Biology of the Cell (I2BC), IBITECS, CEA, CNRS, Univ. Paris-Sud, UPSay, Gif-sur-Yvette cedex, France; 5 AMIBio team, Laboratoire d’informatique de l’École polytechnique (LIX, UMR 7161) / Inria Saclay, UPSay, Palaiseau, France; 6 Laboratoire de Biochimie Théorique, UPR 9080 CNRS Institut de Biologie Physico-Chimique, Paris, France; 7 Sorbonne Université / UPMC, CNRS, IBPS, Laboratoire de Biologie Computationnelle et Quantitative (LCQB), UMR 7238, Paris, France; 8 Institut Universitaire de France; 9 LRI, Univ. Paris-Sud, CNRS, UPSay, Orsay, France; 10 Sorbonne Université / UPMC, Univ. Antilles, Univ. Nice Sophia Antipolis, CNRS, Evolution Paris Seine - Institut de Biologie Paris Seine (EPS - IBPS), Paris, France; 11 Institute for Integrative Biology of the Cell (I2BC), CEA, CNRS, Univ. Paris-Sud, UPSay, Gif-sur-Yvette cedex, France; 12 Department of Structural Biology and CheImistry (CNRS UMR3528) - Center of Bioinformatics, Biostatistics and Integrative Biology (CNRS USR3756) - Structural Bioinformatics Unit, Institut Pasteur, Paris, France; 13 MaIAGE, INRA, UPSay, Jouy-en-Josas, France and LIMSI, CNRS, UPSay, Orsay, France; 14 Sorbonne Université / UPMC, Ecole Normale Supérieure - PLS Research University, Département de Chimie, CNRS, Laboratoire des Biomolécules, UMR 7203 - Univ. Paris Diderot, Sorbonne Paris Cité, Paris, France; 15 Mitochondria, Metals and Oxidative Stress Group, Institut Jacques Monod, UMR 7592, Univ. Paris Diderot, CNRS, Sorbonne Paris Cité, Paris, France; Genome Quebec, CANADA

## Abstract

We present a new educational initiative called Meet-U that aims to train students for collaborative work in computational biology and to bridge the gap between education and research. Meet-U mimics the setup of collaborative research projects and takes advantage of the most popular tools for collaborative work and of cloud computing. Students are grouped in teams of 4–5 people and have to realize a project from A to Z that answers a challenging question in biology. Meet-U promotes "coopetition," as the students collaborate within and across the teams and are also in competition with each other to develop the best final product. Meet-U fosters interactions between different actors of education and research through the organization of a meeting day, open to everyone, where the students present their work to a jury of researchers and jury members give research seminars. This very unique combination of education and research is strongly motivating for the students and provides a formidable opportunity for a scientific community to unite and increase its visibility. We report on our experience with Meet-U in two French universities with master’s students in bioinformatics and modeling, with protein–protein docking as the subject of the course. Meet-U is easy to implement and can be straightforwardly transferred to other fields and/or universities. All the information and data are available at www.meet-u.org.

This is a *PLOS Computational Biology* Education paper.

## Introduction

During the last two decades, biology has been revolutionized by the advent of high-throughput technologies and the rapid increase of computing resources. More and more biological (experimental or computational) data are being produced and made publicly available. This has stimulated the development of collaborative work within and between research teams and of positive competition worldwide (e.g., the DREAM challenge for gene regulatory network reconstruction and the CASP/CAPRI competition for protein and protein complex structure prediction). To keep up with this evolution, we need to devise innovative and adaptative teaching approaches that prepare students for collaborative research work [1].

Examples of pedagogical initiatives promoting collaborative work include hackathons [2] (e.g., using mobile sequencers in bioinformatics education [3]), worldwide competitions like the international Genetically Engineered Machine (iGEM) in synthetic biology [4], and the development of micro- or nanotechnologies (e.g., nanosatellites in space engineering education [5]). The duration of these programs ranges from several hours to a couple of years. They all put the emphasis on hands-on training, which was shown to help students put theoretical concepts into context [6, 7, 8, 9]. The students are evaluated via the attribution of a prize or award or via feedback from the real world (e.g., actual usage of nanosatellites). Competition and confrontation with "harsh reality" [5] stimulate students’ individual investment but can also generate deep disappointment and have detrimental impact on their lives [4].

Here, we propose Meet-U (www.meet-u.org), a new teaching method that combines hands-on training, collaborative work, and project management and aims at bridging the gap between teaching and research. The principle of Meet-U is to make students, grouped in teams of 4–5 members, realize a project from A to Z that adresses an unresolved and open question in biology. One of the original aspects of Meet-U is that a meeting day combining a challenge and a mini-symposium is organized at the end of the course. This event is open to everyone and aims at gathering the scientific community. At this occasion, the students present their methodology and results to a jury of researchers, and the jury members give seminars related to the topic of the course. Meet-U relies on a set of objective metrics to evaluate the students, and both the jury and the teachers contribute to the evaluation. It takes advantage of some of the most popular tools used in research laboratories and companies for version control and collaborative work (Git [https://git-scm.com] and GitHub [https://github.com]). It exploits the resources provided by cloud computing (French Institute of Bioinformatics [https://www.france-bioinformatique.fr/en/cloud]) to deal with the large scales of current biological data.

## Overview of the first edition

**Fig 1:**
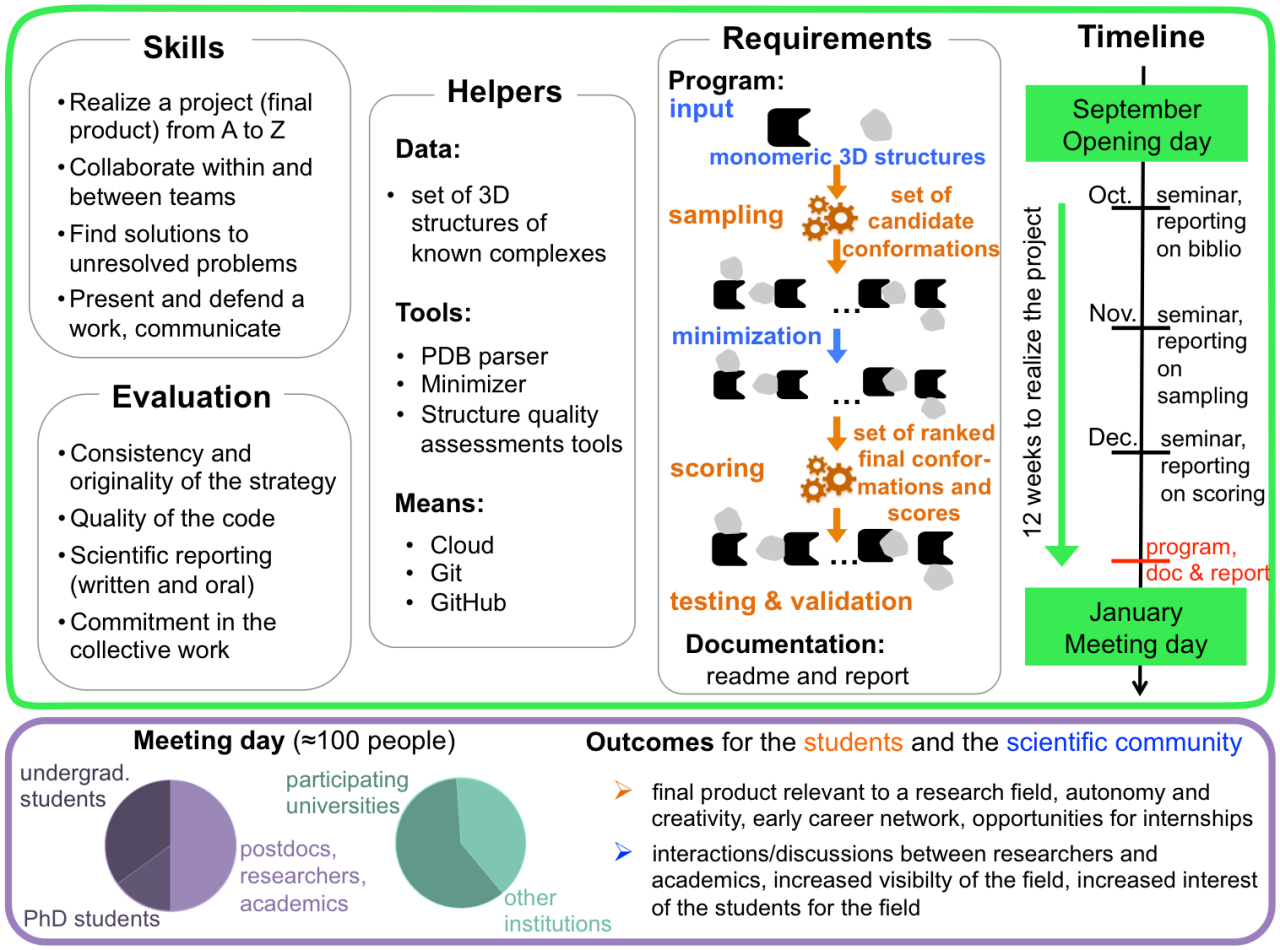
Detailed description of Meet-U. Top green panel: Structure of the course and specifics of the 2016–2017 edition, whose subject was protein–protein docking. In the Requirements section, the data and tools given by the teachers are highlighted in blue, while the tasks and results to be performed by the students are in orange. Schematic representations of the proteins are depicted (receptor in black, ligand in grey). Bottom purple panel: Statistics on the people attending the closing meeting day in January 2017 and outcomes of the course.

**Fig 2:**
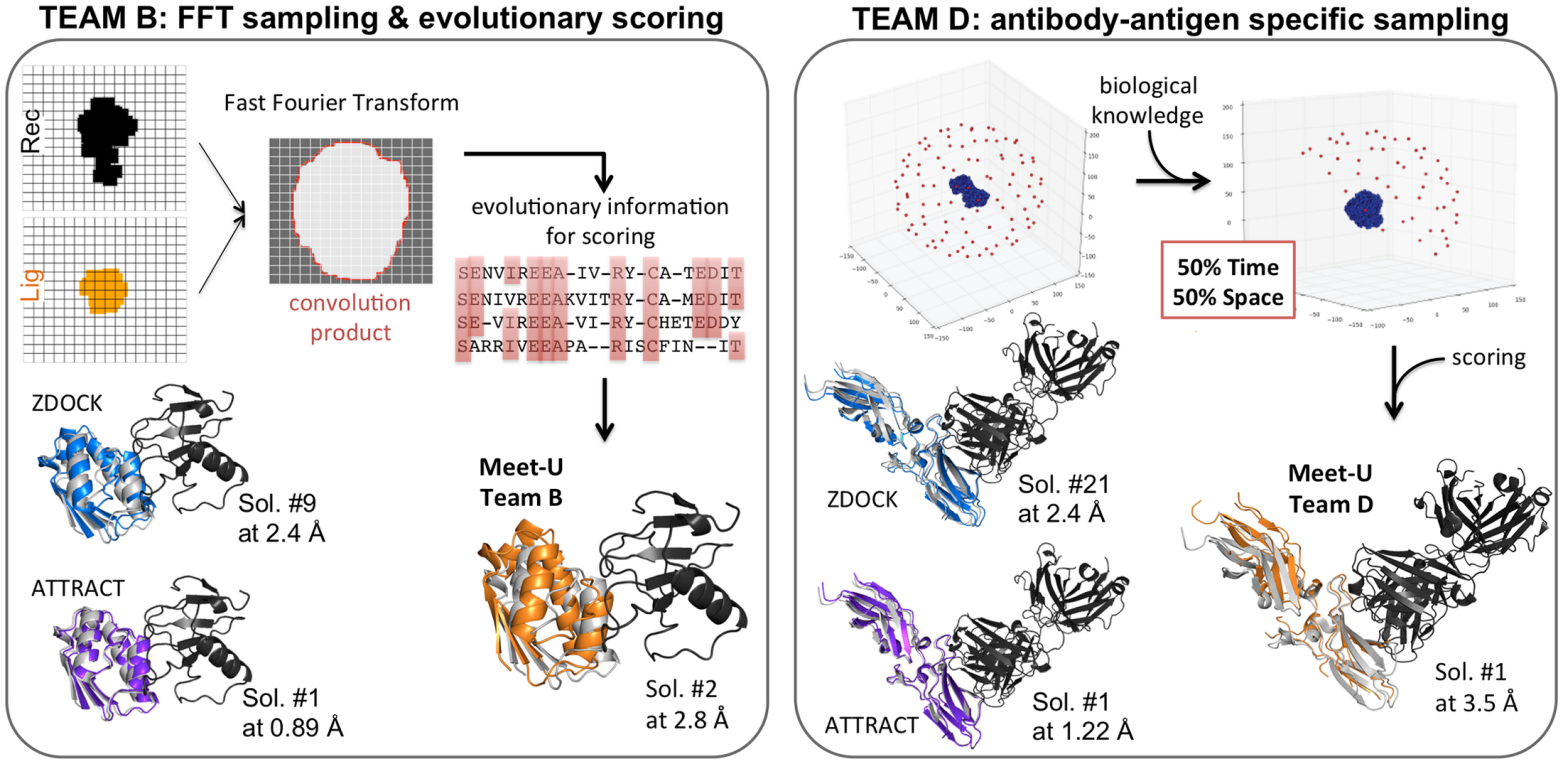
Examples of strategies and results for the 2016–2017 edition. Left panel: Team B implemented an efficient sampling algorithm using a grid representation of the proteins to be docked and FFT. For the scoring, they used evolutionary information extracted from multiple sequence alignments of homologs of the two partners. Right panel: Team D used biological knowledge during the sampling step to filter out conformations early and drastically reduce the search space. The results obtained by the students (Teams B and D) on two complexes (barnase–barstar complex, Protein Data Bank [PDB] code: 1AY7, and an antibody–antigen complex, PDB code: 1JPS, respectively) are comparable to those obtained from state-of-the-art methods, namely ZDOCK [10] and ATTRACT [11]. ZDOCK relies on efficient sampling using FFT and on an optimized energy function [10]. ATTRACT proceeds through minimization steps using an empirical, coarse-grained molecular mechanics potential [11]. Candidate conformations for the complexes are represented as cartoons and superimposed onto the known crystallographic structures. The receptor is in black, the ligand from the candidate conformation is colored (in orange for Meet-U students, blue for ZDOCK, and purple for ATTRACT), and that from the crystallographic structure is in grey. With each candidate conformation are associated its rank, according to the scoring function of the method, and its deviation (in Å) from the crystallographic structure. FFT, Fast Fourier Transform; PDB, protein data bank.

In 2016–2017, Meet-U was implemented at Sorbonne Université/UPMC and Université Paris-Saclay in France. The 28 students who enrolled in the course were studying toward a master’s degree in bioinformatics and were from different backgrounds in biology, computer science, mathematics, and physics. The course is part of the bioinformatics and modeling specialty of the Masters of Computer Science and of Molecular and Cellular Biology at Sorbonne Université/UPMC (http://www.master.bmc.upmc.fr/fr/02_M2/02_BioInfMod/index.php and http://www.lcqb.upmc.fr/BIM/) and of the Masters of Analysis, Modeling and Engineering of Biological and Medical Information at Université Paris-Saclay (http://www.bibs.u-psud.fr/). The topic of the course was structural bioinformatics (more specifically, protein–protein docking). The students had to conceive, design, develop, test, and validate a computational program to predict the three-dimensional (3D) structure of a protein complex, given the structures of the two monomeric partners (Fig 1, top green panel). Prior to the course, they had some basic to intermediate programming experience, and their knowledge in structural bioinformatics ranged from very little to the basics of protein structure determination and modeling. At the end of the course, all 6 teams managed to deliver a finalized end product, and some of the results were comparable to those of state-of-the-art docking tools such as ZDOCK [10] and ATTRACT [11] (Fig 2). The closing meeting day (https://storify.com/pierrepo/colloque-meet-u) gathered about 100 attendees, among whom were the participating students, the pedagogical teams, and some PhD students, researchers, and professors from the greater Paris area (Fig 1, bottom purple panel). Almost half (40%) of the attendees came from institutions other than the two participating universities.

## Aims

Meet-U aims to immerse students into the role of researchers by engaging them in the entire process of a project realization, from the conception to the presentation of the project to their scientific peers. It gives the students the opportunity to address an open question in biology, to experience the pipeline of a bioinformatics research project, and to actively participate in the life of a research community. The question asked, the possible strategies to be implemented (coming from the state-of-the-art), and the data used to validate the students’ algorithms come from the research world and thus are not specifically designed for training. This confers an ambitious character to the project in terms of the complexity of the problem and the scale of the expected results. The course mimics the setup of collaborative research projects, where each participant has a specific field of expertise and is assigned tasks that must be integrated toward the final realization. The meeting day is a very unique opportunity for the students to get their work evaluated by their peers, i.e., researchers who are experts in the field. They do not play the role of students anymore, but they participate in a scientific event, being alternately actors (when they present their work) and spectators (when they listen to the seminars).

## Detailed structure and specifics

The Meet-U course was set as follows (Fig 1, top green panel): it opened with a common 3-hour session, in which all the students met with each other and with the pedagogical team. The teachers gave them a basic knowledge on the topic of the year, namely protein–protein docking, and some good practices for collaborative work and project management (inspired by the “agile management”). The students were asked to form groups of 4–5 members and were encouraged to mix diverse skill sets and backgrounds. Then, they were given about 12 weeks to realize the project, during which 3 sessions of 6 hours were organized every month at each participating university. Each session started with a 1-hour theoretical lecture on a particular aspect of protein–protein docking (evolution and coevolution of the interactions, specificity versus promiscuity of protein interactions, identification of cellular partners at large scale). The rest of the time was dedicated to practical training and exchanges. Each session represented an opportunity to check the advancement of the students by making them report on their work and plans (10–15-minute presentations, intermediate code, or results to show) and to provide technical guidance with their code and/or the tools for version control, collaborative work, and cloud computing. The GitHub platform was used for exchanges within teams, between teams, and with the teachers. Throughout the project, the students had access to a dedicated environment on the academic cloud of the French Institute of Bioinformatics to perform their calculations. About 2 weeks ahead of the closing meeting day, the students were asked to submit their program (executable and sources) along with a documentation and a scientific report. They could then use these 2 weeks to prepare their final presentation.

The project consisted of developing from scratch a rigid-body molecular docking program that took as input the 3D structures of two monomeric partners and returned a list of ranked and scored conformations (3D arrangements) of the complex formed by the two monomers. The principal requirement was to develop the two main steps of the docking procedure: (1) the sampling of the conformational space of the complex and (2) the evaluation of the resulting complex conformations by a scoring function (Fig 1, top green panel). To ease the students’ task, the teachers provided a Python library to handle PDB files and the minimizer procedure used in the docking program MAXDo [12]. The students were free to use or not use it to refine, through energy minimization, the conformations sampled in the first step, prior to scoring. They were also free to choose from among the different state-of-the-art strategies for sampling and scoring or to design a completely new strategy. They were allowed to integrate external tools into their program, as long as the program was able to provide intermediate results for the sampling step (3D structures of candidate conformations) and final results for the scoring step (ordered list of candidate conformations along with computed scores). The material of the course is available at http://meet-u.org/edition_2017.html.

During the closing meeting day, the presentations of the students showed a very good level of quality and a wide diversity of implemented strategies (Fig 2). For example, one team implemented a Fast Fourier Transform (FFT)-based engine for the sampling step and a scoring function including predictions from the tool Joint Evolutionary Trees^2^ (JET^2^) [13], which uses residue conservation, physicochemical, and geometrical properties of protein interfaces (Fig 2, left panel). They obtained very good results on the ribonuclease–barstar complex, with a near-native solution (root mean square deviation [RMSD] = 2.83 Å) ranked as second best (Fig 2, left panel). Several teams used biological constraints to reduce the docking space search, particularly for the prediction of antibody–antigen 3D assemblies. One team implemented an algorithm to filter out irrelevant conformations early using predictions of antigen binding sites from a sequence-based method [14]. This reduced the search space and time by about 50% (Fig 2, right panel). The presentations of the jury members (45 minutes each) showed recent developments in protein–protein docking methods and examples of applications, some of them beyond the scope of "classical" molecular docking (e.g., protein cellular partner identification and discrimination and experimental structure reconstruction using elastic network models).

## Acquired skills and outcomes

Students who engage in Meet-U acquire skills necessary to develop and manage research projects in academic or industrial environments and that are particularly needed in bioinformatics/computational biology. They learn how to apply their theoretical knowledge, search in the literature, program, collectively conduct a project, look for solutions to technical or conceptual problems on their own, report on their work publicly, and exploit cloud computing resources (Fig 1, top green panel). These skills are generally difficult to transmit in classical classrooms. Moreover, one of the values of Meet-U is that, although the biological question and the project requirements are (and must be) well specified, the students are free to choose from many possible strategies and are encouraged to propose original ideas and take some risks. Thus, they learn how to formulate their own ideas, turn them into concrete realizations, and argue with their mates to defend their point of view and define a common strategy. As no hierarchy is imposed on the teams and no direct instructions are given, the students within each team have to invent a way of working and, more importantly, "functioning" together. Coming from different backgrounds, they integrate their different ways of thinking and languages and develop a critical mindset. Biomolecules such as proteins, because they are complex biological objects, are particularly amenable to inspiring a wide range of points of views and approaches, and indeed we observed that students from different backgrounds proposed very different strategies. Finally, the whole course and particularly the final meeting offer the students a unique opportunity to start building their career network with the other students and with teachers and researchers as well as to prospect for internships (Fig 1, bottom purple panel).

For the research community, Meet-U is an occasion for exchanging on a common topic, becoming collectively aware of the "local forces" and increasing its visibility in the participating universities and beyond (Fig 1, bottom purple panel). Especially in a highly interdisciplinary field like computational biology, it is very important to create such occasions where researchers from different backgrounds working on the same subject can interact. Meet-U also provides a way to draw some attention to a particular discipline and increase the students’ interest in it. For example, the 2016–2017 edition motivated 5 students (among 28) to look for internships and doctorates in structural bioinformatics, which was significantly more than in previous years. Meet-U is intended to cover some (all will be impossible) of the various topics of computational biology. Organizing the course over several years on different subjects should stimulate exchanges between the different disciplines of computational biology, which will be in turn highly beneficial for our research. Already, the 2016–2017 issue has attracted researchers and teachers from sequence analysis, genomics, and biostatistics.

## Evaluation of the first edition

To evaluate Meet-U’s first edition, we relied on students’ activity, feedback, and evolution and on teachers’ and researchers’ perception of the experience. Overall, the students gave very positive feedback. They insisted on the fact that the course was unique in their formation with respect to several aspects: collaborative work, autonomous bibliographical research, realization of a final functional product with well-defined specifics/requirements, usage of the cloud, meeting with students from another institution, and feelings of being in direct contact with the teachers and with researchers. They noted that it was important for them to be able to choose their teammates and to be able to see each other and talk about the project on a daily basis. They recognized that the course required a substantial amount of personal work.

We evaluated the effectiveness of several elements of Meet-U in bringing valuable new educational contributions compared to traditional teaching methods and identified some weaknesses that could be improved in the next edition. First, the team format, the usage of GitHub, and the final meeting day resulted in a form of "coopetition" (positive competition). The different teams were stimulated to compete with each other to realize the best product and present the best results to the jury and the public. The GitHub platform was instrumental in facilitating and stimulating collaboration within and between teams. In particular, the GitHub forum, in which students could publicly post questions and comments relative to the project, created a collective dynamic. We observed that students coming from different teams helped each other by solving technical issues and suggesting improvements. This collaborative aspect between teams was very beneficial and could have been further managed to ensure productive work. To this end, we decided to formally make different teams realize a part of the project together in the next edition (see below). Second, the combination of an ambitious open question with the requirement to deliver a finalized end product and present it publicly had a positive impact on students’ perception of research and on the way they could imagine themselves playing a role in it. These elements were powerful in demystifying research and especially the project topic. Several students reported that they gained self-confidence and autonomy in the process and that the Meet-U experience inspired them to pursue a career in research by doing a PhD. Third, both Git/GitHub and the computing cloud gave the students some independence with respect to local resources and provided homogeneous access within each group to test and debug ongoing developments, mitigating the risk of overspecialization. Particularly with the computing cloud, they had access to a properly configured virtual environment and to an amount of computing power enabling them to test and parameterize their program with their own benchmark and to actually produce results beyond simple validation. Several teams applied their programs on several dozen cases, even though the execution time required for the prediction of a single complex was substantial (between 1 and 16 hours). Finally, Meet-U’s "open" and participative format (several teachers elaborating the course, continuous exchanges between all participants via GitHub, and the final meeting that included a jury of researchers and was open to the public) enabled them to develop a multiplicity of points of view on the experience, which favored the emergence of new ideas and the rapid adaptation of the course (see below). In practice, Meet-U involved several pedagogical challenges (e.g., finding a compromise between maintaining students’ motivation and keeping up with the ambition of the project, ensuring a positive experience for students whatever their results, and making students aware of what they had accomplished and acquired) to which teachers proposed solutions collectively. For them, this collaborative framework (compared to the one-teacher traditional class) proved very stimulating and fostered fruitful exchanges across different institutions concerning education and research.

## Changes for the next edition

With the double motivation of alleviating the workload and further stimulating collaboration between teams, we divided the project into two steps for Meet-U’s second edition. About half of the teams are in charge of the sampling step, while the other half deal with the scoring step. We added a common meeting day halfway through the course, in which each team presents its strategy, program, and results to all other teams and looks for a partner team. Teams must pair up to integrate their respective programs into the finalized end product: a complete docking program performing docking and scoring. In addition, thanks to the success of the first edition, a third university joined the second edition, and the number of participating students increased by 60%. We estimate that we are close to the limit where we can keep the unity of the course and organize a single closing meeting day.

## How to Meet-U?

Meet-U is easily transferrable to any university or group of universities interested in the concept. We recommend organizing the course over a period of 10 to 15 weeks, during which teachers and students meet each other on 4 to 7 occasions at regular time intervals. The required prior knowledge should be minimal so that any student in bioinformatics can enroll in the course. Nevertheless, we recommend that a significant portion of the students have some basic prior knowledge in the discipline related to the project topic. If this is not the case, introductory lectures and/or practicals should be organized before the beginning of the project. During the opening session (2–3 hours), the teachers should provide the basic concepts associated with the project topic and detailed specifications of the project so that students can start working right away. It is also important to give the students some good practice at project management and collaborative work at this stage. The session can be extended by one hour or so to give information about how to perform literature searches on the project topic. Following this opening session, the students meet the teachers regularly (every 2–3 weeks) and present their advancement to them. Typically, one teacher for two teams is a good ratio. The teachers check that all the teams progress at a reasonable pace and that the strategies and choices of implementation are sound. They guide the students, help them find solutions, and provide technical guidance. They also provide information on how to use the tools dedicated to collaborative work and cloud computing and advise students on how to efficiently search through the bibliography and conduct a project. These meeting sessions also include theoretical lectures of one hour each intended to help students understand the application-specific issues associated with the project, inspire them by describing some state-of-the-art approaches, and give them a broader perspective on the subject. In total, the students spend 15–20 hours in class with the teachers. They must also dedicate a substantial amount of personal time to the realization of the project.

To further help the students, the teachers can provide them libraries, helper tools, and biological datasets for tests and validation. They may also invest some time to set up a properly configured virtual environment on the computing cloud, including helper tools. Considering the observed benefits of the cloud, such an investment is definitely worth it.

At the end of the course, the students must provide a written report of about 10–15 pages that presents the subject, the developed method, and the obtained results. They should also provide a one- or two-page digest of the report to summarize key results of their project. They also give their commented code and output files for test cases. All this material should be given to the pedagogical team and the jury members at least 2 weeks before the closing meeting day. On that day, the students present their work in the morning to the members of the jury and the audience. A typical format is 10–15 minutes of presentation and 10 minutes of questions. The jury deliberates during the lunch break with the pedagogical team, which will in turn determine the ranking/grades of the students. The final evaluation is based on several objective criteria, including the quality of the implementation, the relevance and innovative character of the chosen strategy, the quality of the obtained results, the quality of the presentation, and the quality of the written report. The participation/involvement in the project is also assessed via the activity on the version control system, the observed behavior during lectures, and the appreciation by the jury. In the afternoon, the members of the jury give seminars on their recent research work related to the topic.

Meet-U can be implemented within one department of one university, but we strongly recommend taking this opportunity to create a collaborative course between several universities and departments. We recommend forming each team with students belonging to the same institution to facilitate exchanges between them. Collaboration between teams may be spontaneous, suggested, or imposed. This latter option may permit them to realize more ambitious projects. The opening sessions and the final meeting days require the presence of all students engaged in the course. A common day can also be organized halfway through the course, especially if teams involving different departments/universities have to share and integrate their respective programs.

We recommend formally recognizing Meet-U as part of the students’ official academic curriculum and dedicating a significant amount of credits to it. It is important that the personal time students have to invest in Meet-U is properly taken into account in their curriculum to prevent unequal participation. Although most evaluation criteria are defined on a team basis, students should be evaluated individually and made aware of this fact to avoid investment imbalance within teams.

To properly set up the course, the pedagogical team should comprise at least two teachers from each participating university, including one specialist on the project topic. Time must be invested to prepare the course material and to answer students’ questions on the forum, in addition to face-to-face time. Ideally, students may have free access to an academic computing cloud (as was the case here with the IFB cloud). Alternatively, a budget should be dedicated to computing resources or the project should be adapted. Financing is required for the closing meeting day for food and drinks (lunch and coffee breaks, cocktails) and for the trip and accommodation expenses of the jury members, if needed.

Finally, we encourage institutions interested in the concept to implement it and provide us feedback on their experience of Meet-U by directly contacting us and completing the online form at http://www.meet-u.org/howto.html. This will enable us to compare different editions of Meet-U on different topics and in different contexts, which shall be very beneficial for the whole community.

## Perspectives of extension

The setup of the course enables us to completely renew the topic and the people every two years or so. Depending on the level of expertise and maturity of the students and on the chosen theme/discipline, the teachers may adapt the levels of complexity and specificity of the problem. In the case presented here, the students were asked an already well-specified question. Alternatively, the students may be asked to formulate, test, and refine their own research questions and hypotheses for an even more "immersive" experience into research. In that context, the overall duration of the course should be extended so that students can perform extensive bibliographical research, debate between themselves, establish an elaborated strategy, and possibly modify it depending on the obtained results. The requirement for a finalized end product to be publicly presented should nevertheless be maintained, as the concrete realization of a functional and useful final product is one of the key elements motivating students and guaranteeing Meet-U’s success. Another perspective would be to pair up Meet-U with other training programs, particularly in wet biology, which would provide the question and/or data for the project. These two aspects may be combined and are likely to boost students’ creativity.

The strength of Meet-U is that the themes and the people renew, ensuring its constant evolution, while the overarching concept remains.
